# Rapid resistance profiling of SARS-CoV-2 protease inhibitors

**DOI:** 10.1038/s44259-023-00009-0

**Published:** 2023-08-20

**Authors:** Seyed Arad Moghadasi, Rayhan G. Biswas, Daniel A. Harki, Reuben S. Harris

**Affiliations:** 1 University of Minnesota, Minneapolis, 55455 MN USA; 2 Department of Biochemistry and Structural Biology, University of Texas Health San Antonio, San Antonio, 78229 TX USA; 3 Howard Hughes Medical Institute, University of Texas Health San Antonio, San Antonio, 78229 TX USA

**Keywords:** Antivirals, Antiviral agents

## Abstract

Resistance to nirmatrelvir (Paxlovid) has been shown by multiple groups and may already exist in clinical SARS-CoV-2 isolates. Here a robust cell-based assay is used to determine the relative potencies of nirmatrelvir, ensitrelvir, and FB2001 against a panel of SARS-CoV-2 main protease (M^pro^) variants. The results reveal that these three drugs have at least partly distinct resistance mutation profiles and raise the possibility that the latter compounds may be effective in some instances of Paxlovid resistance and *vice versa*.

Antiviral drugs are necessary to combat SARS-CoV-2/COVID-19, particularly with waning interest in the repeated vaccination boosts necessary to keep-up with virus evolution. The main protease (M^pro^) of SARS-CoV-2 is essential for virus replication and, accordingly, a proven therapeutic target as evidenced by Paxlovid, an orally administered antiviral drug (active component: nirmatrelvir; Fig. 1a). However, as for drugs developed to treat other viruses^1^ and for first-generation SARS-CoV-2 vaccines, there is a high probability that variants will emerge that resist nirmatrelvir. Indeed, a flurry of recent studies has described a variety of candidate nirmatrelvir-resistance mutations^2–9^. Thus, considerable urgency exists to develop next-generation M^pro^ inhibitors with different resistance mechanisms and, in parallel, robust systems to rapidly assess the potential impact of candidate resistance mutations. Two additional M^pro^ inhibitors, Ensitrelvir (Xocova) and FB2001 (Bofutrelvir), which are orally and intravenously administered, respectively, are currently being evaluated in clinical trials^10,11^ (Fig. 1a). Ensitrelvir has also received EUA in Japan (https://www.shionogi.com/global/en/news/2022/11/e20221122.html). However, the potency of these drug candidates against nirmatrelvir-resistant M^pro^ variants has yet to be fully assessed.

**Fig. 1:**
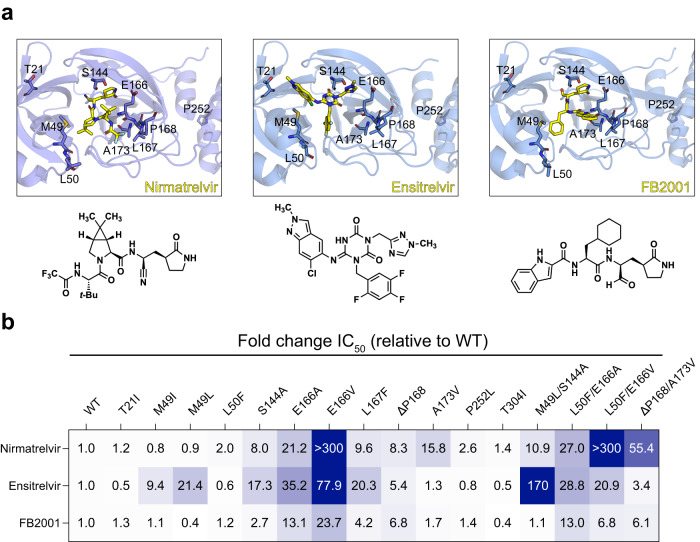
Resistance of M^pro^ variants to nirmatrelvir, ensitrelvir, and FB2001. **a** Co-crystal structures of SARS-CoV-2 M^pro^ in complex with nirmatrelvir (PDB:7SI9), ensitrelvir (PDB:7VU6), or FB2001 (PDB:6LZE) (chemical structures depicted below each image). Labeled residues, as well as T304I, are tested in panel B. **b** Fold-change in IC_50_ relative to WT for the indicated mutants using the live cell gain-of-signal assay in 293T cells.

We recently developed a gain-of-signal system for facile quantification of M^pro^ inhibition^12^, and used it together with evolution- and structure-guided approaches to characterize candidate nirmatrelvir- and ensitrelvir-resistance mutations^2^. This system is comprised of a single plasmid encoding SARS-CoV-2 M^pro^ flanked by its native cleavage sites and an N-terminal myristoylation domain from Src kinase and a C-terminal HIV-1 Tat fused to firefly luciferase^12^ (schematic in Supplementary Fig. 1a). Overexpression of catalytically active M^pro^ in this system results in cleavage of multiple cellular substrates^13,14^ including at least one required for RNA Polymerase II-dependent gene expression^12^, which results in low luciferase signal that can be restored by either genetic inactivation or chemical inhibition^2,12^.

Here, this system is used to examine an expanded panel of M^pro^ single and double mutants based on recent studies by our group and others^2–9^ and determine their impact on the potency of nirmatrelvir, ensitrelvir, and FB2001 (heatmap of results in Fig. 1b; specific references and quantification summary in Table 1; representative dose responses in Supplementary Fig. 1b). Several single amino acid substitution mutants selected during serial passaging^3,5–7^ including T21I, L50F, P252L, and T304I show minimal resistance to nirmatrelvir, ensitrelvir, or FB2001 in our assay. In contrast, selective resistance to ensitrelvir is conferred by M49I and M49L, and selective resistance to nirmatrelvir by A173V (highlighted in gray in Table 1). ∆P168 elicits similar resistance to all inhibitors, and synergistic resistance to nirmatrelvir when combined with A173V. S144A and L167F show the greatest resistance to ensitrelvir, intermediate resistance to nirmatrelvir, and lower resistance toward FB2001. M49L in combination with S144A elicits very high resistance to ensitrelvir, intermediate resistance to nirmatrelvir, and essentially no resistance to FB2001. In contrast to E166A and L50F/E166A, which cause a similar broad-spectrum resistance, E166V and L50F/E166V elicit very high resistance to nirmatrelvir, intermediate resistance to ensitrelvir, and substantially lower resistance to FB2001.

**Table 1:** IC_50_ values of nirmatrelvir, ensitrelvir, and FB2001 against M^pro^ resistance variants.

M^pro^ variant [ref(s)]	IC_50_ [nM] (Fold-change relative to WT)			Mutant identification
	Nirmatrelvir	Ensitrelvir	FB2001	
WT	29.4 (1.0)	35.9 (1.0)	27.2 (1.0)	–
T21I^3,5^	36.0 (1.2)	16.3 (0.5)	34.0 (1.3)	Serial passage
M49I^2,8^	23.0 (0.8)	**338 (9.4)**	29.8 (1.1)	Naturally occurring
M49L^2^^,link below^	27.1 (0.9)	**769 (21.4)**	10.7 (0.4)	Naturally occurring
L50F^3,5,6^	58.4 (2.0)	21.0 (0.6)	33.2 (1.2)	Serial passage
S144A^5^^,link below^	236 (8.0)	623 (17.3)	74.7 (2.7)	Serial passage
E166A^5,6^	622 (21.2)	126 (35.2)	355 (13.1)	Serial passage
E166V^3,5^	>10,000 (>300)	2800 (77.9)	645 (23.7)	Serial passage
L167F^5–7^	282 (9.6)	728 (20.3)	115 (4.2)	Serial passage
∆P168^2^	243 (8.3)	193 (5.4)	184 (6.8)	Naturally occurring
A173V^2,5^	**460 (15.8)**	45.9 (1.3)	45.7 (1.7)	Naturally occurring
P252L^5^	76.9 (2.6)	28.8 (0.8)	38.9 (1.4)	Serial passage
T304I^3,5^	40.7 (1.4)	19.0 (0.5)	10.5 (0.4)	Serial passage
M49L/S144A^This study, link below^	321 (10.9)	6110 (170)	29.0 (1.1)	Rational combination
L50F/E166A^5,6^	793 (27)	1040 (28.8)	355 (13)	Serial passage
L50F/E166V^3,5^	>10,000 (>300)	751 (20.9)	185 (6.8)	Serial passage
∆P168/A173V^2^	1630 (55.4)	122 (3.4)	166 (6.1)	Rational combination

Clear examples of single amino acid substitution mutations conferring selective resistance to nirmatrelvir and ensitrelvir are in bold; similarly selective mutations have yet to be found for FB2001. The relative values in brackets were used for the heatmap in Fig. 1b.

https://www.pmda.go.jp/drugs/2022/P20220719001/340018000_30400AMX00205000_H102_2.pdf.

Genetic mutants can also exhibit phenotypes in our system in the absence of drug, with some showing wildtype M^pro^ activity (background luminescence) and others compromising activity weakly or strongly depending on the nature of the mutation (low to high luminescence, respectively). For example, in comparison to wildtype M^pro^, catalytic mutants such as C145A yield 50- to 100-fold higher luminescence^2,12^. The M^pro^ variant constructs used here display a range of luminescence levels in the absence of drug indicative of near-normal M^pro^ activity (notably, M49I and M49L), weakly compromised M^pro^ activity (notably, A173V), and strongly compromised M^pro^ activity (notably, E166V) (Supplementary Fig. 2). These results suggest that several variants can confer at least partial drug resistance with little loss in M^pro^ functionality (and likely also viral fitness), whereas others such as E166V require suppressor mutations such as L50F to restore M^pro^ function to a level that enables virus replication (evidenced by recent resistance studies with pathogenic SARS-CoV-2 in culture and *in vivo* in animal models^3,5^).

The results here demonstrate that nirmatrelvir, ensitrelvir, and FB2001 have distinct susceptibilities to several resistance-associated substitutions, even without knowledge of the precise details of each molecular mechanism. Importantly, in cases where *bona fide* resistance develops against one drug, the others may still prove effective depending on the identity of the selected mutations. Although FB2001 has structural similarity to nirmatrelvir, it appears less susceptible to several resistance-associated mutations, which suggests that it and potentially other peptidomimetic inhibitors in development may be able to achieve greater durability. In support of this possibility, FB2001 also exhibits efficacy against the distantly related coronaviruses 229E and NL63 compared to nirmatrelvir and ensitrelvir (Supplementary Fig. 3a, b). However, the antiviral activity of each drug against large panels of diverse viruses, as well as dedicated resistance studies, will be required to fully evaluate broader spectrum utility.

Importantly, the relative fold-resistance as determined by the gain-of-signal assay associates positively with published findings using infectious viruses^2,3,5–7^ (Supplementary Fig. 4). This live cell assay therefore provides an accurate, safe, and rapid system for assessing resistance. As the SARS-CoV-2 variant pool deepens, this assay and variant panel can be expanded in lock-step to provide early resistance “fingerprints” of candidate next-generation M^pro^ inhibitors. Such an early profiling strategy has the potential to minimize the risks of developing drugs prone to cross-resistance and to help identify inhibitors with the highest barriers to resistance and broadest spectrum of utility.

## Methods

### DNA constructs and cell culture

The live cell gain-of-signal assay for M^pro^ inhibition was performed using the pcDNA5/TO-Src-M^pro^-Tat-fLuc reporter construct which encodes the N-myristoylation domain of Src kinase followed by SARS-CoV-2 WA1 M^pro^ flanked by its cognate cleavage sites, HIV-1 Tat and firefly luciferase^12^. All SARS-CoV-2 M^pro^ single and double mutants selected for analysis here were based on recent reports of candidate resistant mutants^2–9^ generated by site-directed mutagenesis (primers in Supplementary Table 1) and verified by Sanger sequencing. Transfections were done using 293T cells (ATCC CRL-3216) maintained at 37 °C and 5% CO_2_ in DMEM (Gibco catalog number 11875093) supplemented with 10% fetal bovine serum (ThermoFisher catalog number 11965084) and penicillin-streptomycin (Gibco catalog number 15140122).

### M^pro^ resistance experiments

For each individual SARS-CoV-2 M^pro^ variant, 3 × 10^6^ 293T (ATCC CRL-3216) cells were plated in a 10 cm dish and transfected 24 h later with 2 µg of the corresponding variant plasmid using TransIT-LT1 (Mirus catalog number MIR 2304). Transfected cells were incubated at 37 °C and 5% CO_2_ for 4 h, washed once with phosphate buffered saline (PBS), trypsinized, resuspended in fresh media, and diluted to a concentration of 4 × 10^5^ cells/ml. 50 µL of each cell suspension was added to a 96-well white clear bottom cell culture plate (ThermoFisher #165306) containing pre-aliquoted inhibitor-supplemented media for a final concentration of 20,000 cells per well and inhibitor dose response range of 10 µM to 2.4 nM. Inhibitors were purchased from commercial vendors (nirmatrelvir, MedChemExpress catalog number HY-138687; ensitrelvir, MedChemExpress catalog number HY-143216; FB2001, Sigma-Aldrich catalog number SML2877) and purity was confirmed by HPLC and NMR. After an additional 44 h incubation (48 h total post-transfection), luciferase activity was quantified by removing growth medium and adding 50 µL of Bright-Glo reagent (Promega catalog number E2610) to each well and incubating at room temperature in the dark for 2 m before measuring luminescence on a Biotek Synergy H1 plate reader.

Percent M^pro^ inhibition was calculated at each concentration of inhibitor using the formula below using the relative luminescence of an inhibitor (RLi) treated sample to the untreated control for each individual mutant.

1 $$\% {inhibition}=100-(100/{RLi})$$

Results were plotted using GraphPad Prism 9 and fit using a four-parameter non-linear regression to calculate IC_50_ values (Fig. S1; Table 1). Resistance of mutants was calculated by the fold change in IC_50_ of the mutant relative to WT M^pro^, and these values were used to generate a heatmap in GraphPad Prism 9 (Fig. 1b).

As an increase in luminescence in the absence of any inhibitor treatment is indicative of decreased M^pro^ catalytic activity, the relative activity of each mutant was calculated by the formula below using the relative luminescence of a mutant (RLm) to the WT enzyme in the absence of inhibitor (Fig. S2).

2 $$\% {activity}=100-(100/{RLm})$$

### Reporting summary

Further information on research design is available in the Nature Research Reporting Summary linked to this article.

## Supplementary information

Supplemental Material

REPORTING SUMMARY

## Data Availability

All results are presented in the main display items or supplementary figures. The SARS-CoV-2 M^pro^ gain-of-signal plasmid and mutant derivatives are available upon email request to rsh@uthscsa.edu and completion of a MTA (U.S. Provisional Application Serial No. 63/108,611, filed on November 2, 2020).
