# Supplementary material for: Rapid resistance profiling of SARS-CoV-2 protease inhibitors
Source: NPJ Antimicrob Resist. 2023 Aug 20;1:9. doi: 10.1038/s44259-023-00009-0 (PMC11721111; doi:10.1038/s44259-023-00009-0)
Supplement: Supplementary file 1 — Supplemental Material [file 44259_2023_9_MOESM1_ESM.pdf]

Online Supplementary Information for:

Rapid resistance profiling of SARS-CoV-2 protease inhibitors

Seyed Arad Moghadasi[1], Rayhan G. Biswas[1], Daniel A. Harki[1] & Reuben S. Harris[2,3]

[1] University of Minnesota, Minneapolis, Minnesota, USA

[2] Department of Biochemistry and Structural Biology, University of Texas Health San Antonio, San Antonio, Texas, USA, 78229

[3] Howard Hughes Medical Institute, University of Texas Health San Antonio, San Antonio, Texas, USA

Correspondence: rsh@uthscsa.edu

Contents: Supplementary Figures 1-4 and Supplementary Table 1

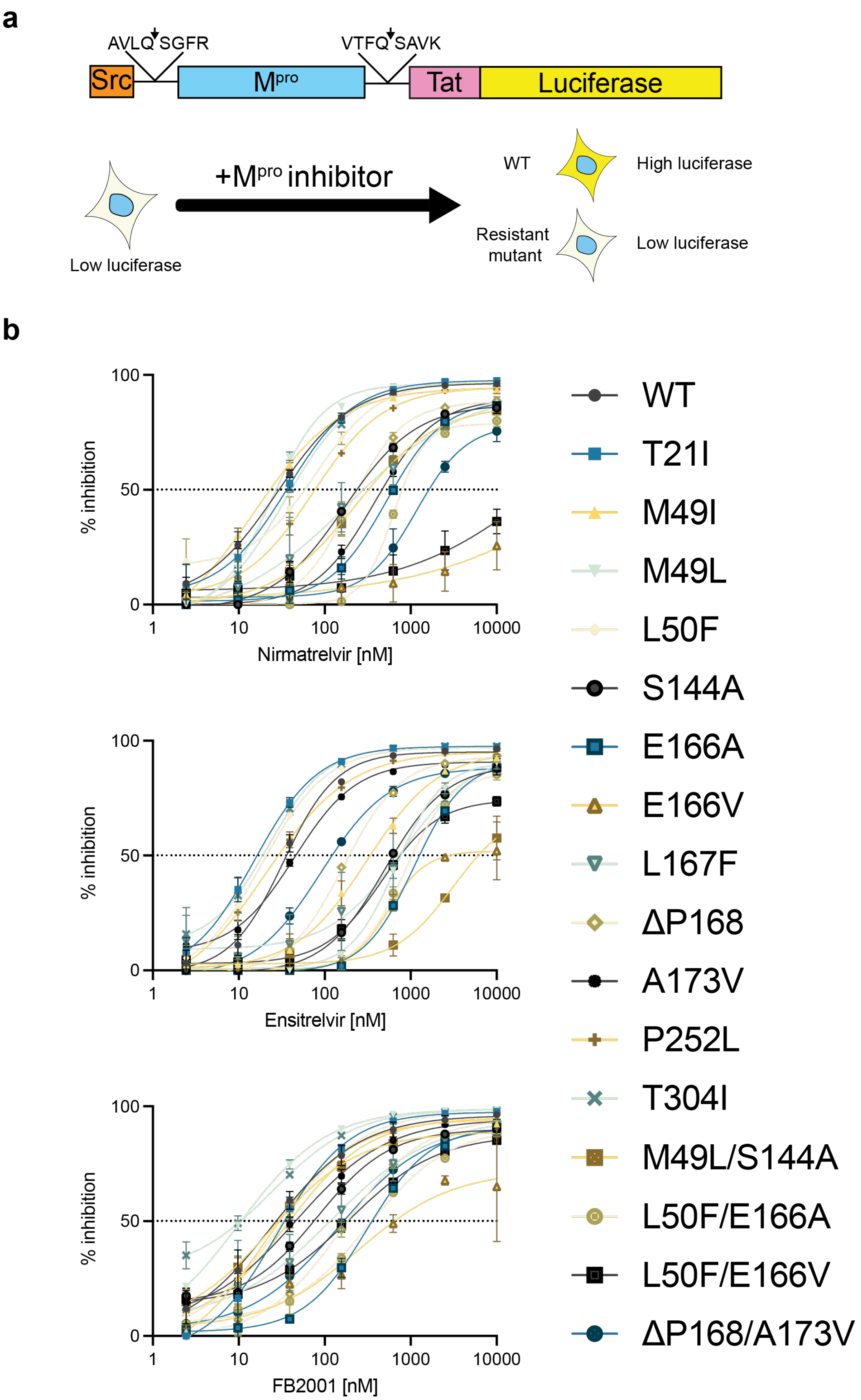
a
AVLQ SGFR
VTFQ SAVK
Src
Mpro
Tat
Luciferase
+Mpro inhibitor
Low luciferase
WT
High luciferase
Resistant mutant
Low luciferase
b
100
50
0
% inhibition
1
10
100
1000
10000
Nirmatrelvir [nM]
Ensitrelvir [nM]
FB2001 [nM]
WT
T21I
M49I
M49L
L50F
S144A
E166A
E166V
L167F
ΔP168
A173V
P252L
T304I
M49L/S144A
L50F/E166A
L50F/E166V
ΔP168/A173V

Supplementary Figure 1. Dose response curves showing inhibition of WT and mutant M^{pro} enzymes by nirmatrelvir, ensitrelvir, and FB2001.

(**a**) Schematic of Src-M^{pro}-Tat-fLuc assay in which transfection of a catalytically active WT M^{pro} construct into 293T cells yields low luciferase expression due to cleavage of host substrates that prevent reporter expression. Inhibition of M^{pro} catalytic activity by chemical (shown) or genetic methods results in quantifiable increases in luminescent signal.

(**b**) Dose responses of M^{pro} variants using the gain-of-signal assay in cells treated with indicated inhibitors in a 4-fold serial dilution beginning at 10μM (data are mean +/- s.d. of biologically independent triplicate experiments). IC_{50} values for each inhibitor are listed in Table 1.

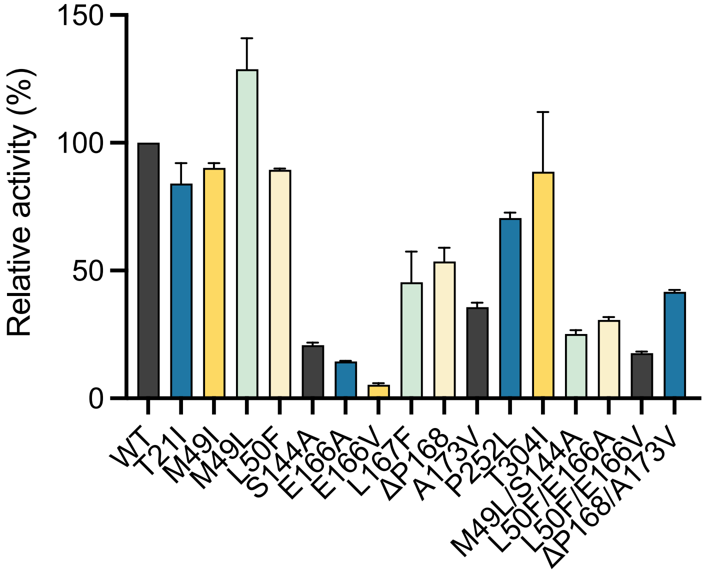

Supplementary Figure 2. Relative activity of M^{pro} mutants. A histogram showing the relative catalytic activity of each M^{pro} mutant relative to the WT construct (data are mean +/- s.d. of biologically independent triplicate experiments, normalized to 100% to facilitate comparison). Several single mutants such as T21I, M49I, M49L, L50F, and T304I show near WT activity. Other mutants such as A173V show modest 1.5 to 3-fold decreases in relative activity, and a few such as E166V are severely compromised. L50F partly restores the activity of E166A and E166V mutants consistent with prior reports[2-9].

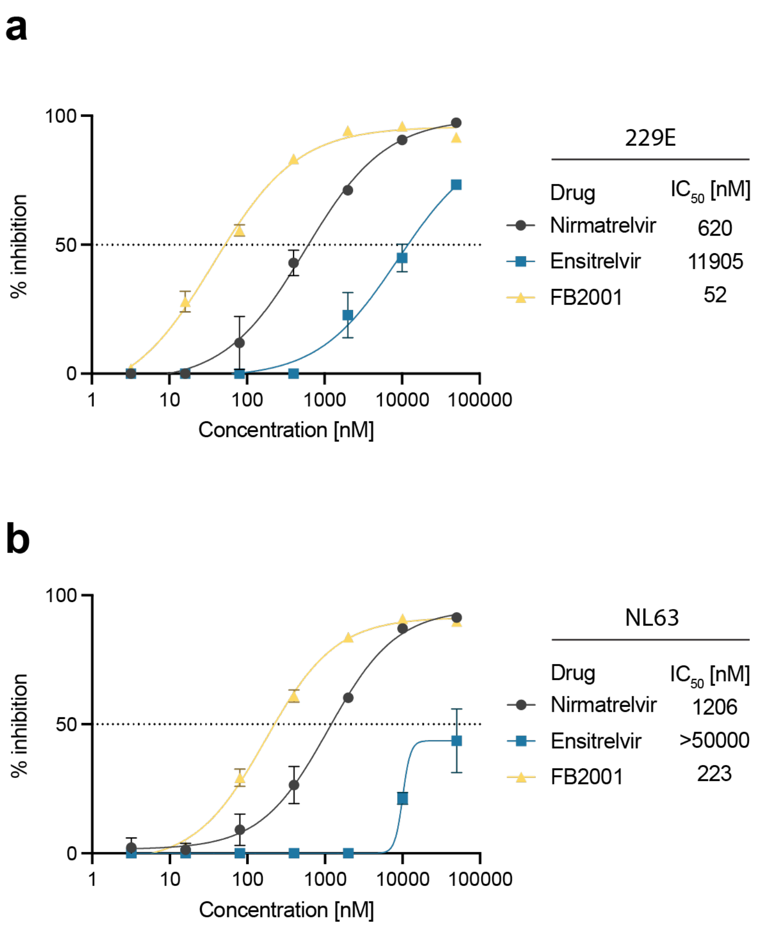

Supplementary Figure 3. Dose response curves showing inhibition of the alphacoronaviruses 229E and NL63 M^{pro} by nirmatrelvir, ensitrelvir, and FB2001. (**a-b**) Dose response of 229E and NL63 M^{pro} proteins using the gain-of-signal assay in cells treated with indicated inhibitors in a 4-fold serial dilution beginning at 50µM (data are mean +/- s.d. of biologically independent triplicate experiments).

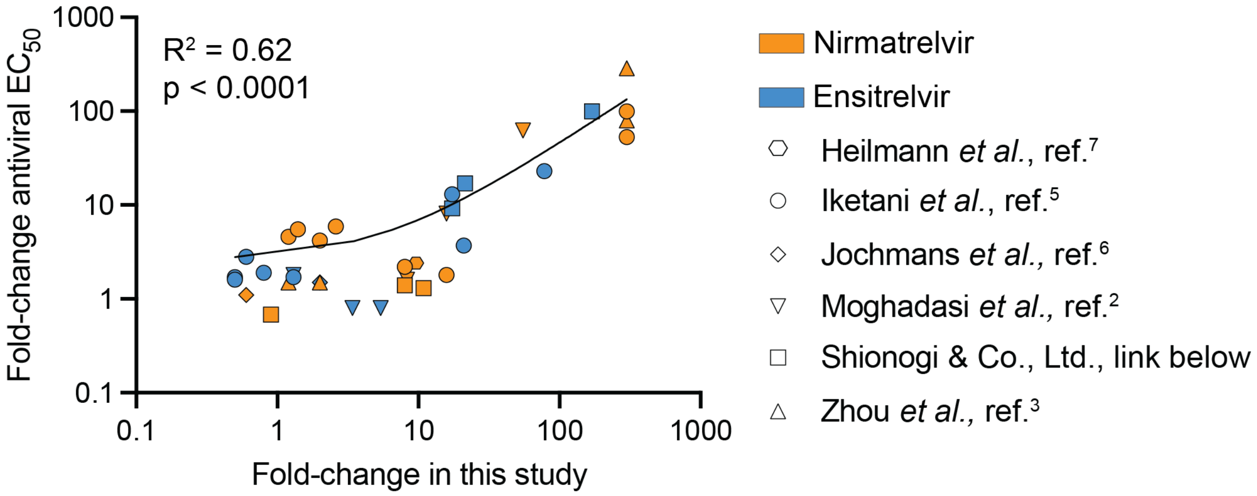

Supplementary Figure 4. Positive association between M^{pro} inhibitor resistance determined using infectious SARS-CoV-2 (published studies) and the gain-of-signal system (this study). A dot-plot showing the positive association between the fold-change in antiviral EC_{50} as determined with infectious SARS-CoV-2 (refs.[2,3,5-7] and https://www.pmda.go.jp/drugs/2022/P20220719001/340018000_30400AMX00205000_H102_2.pdf) and the fold-change in IC_{50} as determined by the gain-of-signal assay (this study). Data were analyzed by a simple linear regression and calculation of Pearson Correlation coefficient (r) in GraphPad Prism 9. Orange and blue shaded symbols represent data with nirmatrelvir and ensitrelvir, respectively. FB2001 resistance mutants have yet to be reported.

Supplementary Table 1. Site directed mutagenesis primers for generating M^{pro} variants.

Primer name	Primer sequence
T21I forward	GTATGGTCCAAGTAATATGTGGTACCACTACCCTTAATGG
T21I reverse	GTAGTGGTACCACATATTACTTGGACCATACATCCTTCAAC
M49I forward	GCACTAGTGAGGATATTCTTAATCCCAATTACGAAGACC
M49I reverse	GTAATTGGGATTAAGAATATCCTCACTAGTGCAGATTACGTG
M49L forward	ACTAGTGAGGATCTACTTAATCCCAATTACGAAGACCTTTTG
M49L reverse	GTAATTGGGATTAAGTAGATCCTCACTAGTGCAGATTAC
L50F[3,5,6]	CTAGTGAGGATATGTTCAATCCCAATTACGAAGACCTTTTGATTCG
L50F reverse	CGTAATTGGGATTGAACATATCCTCACTAGTGCAGATTACG
S144A forward	CTGAACGGCGCATGCGGTTCCGTTG
S144A reverse	GAACCGCATGCGCCGTTCAGAAATGAACCC
E166A[5,6]	CACCATATGGCACTCCCTACCGGTGTC
E166A reverse	GTAGGGAGTGCCATATGGTGCATGTAGC
E166V forward	CACCATATGGTACTCCCTACCGGTGTC
E166V reverse	CGGTAGGGAGTACCATATGGTGCATG
L167F forward	CCATATGGAATTCCCTACCGGTGTCC
L167F reverse	CCGGTAGGGAATTCCATATGGTGCATG
ΔP168 forward	TATGGAACTCACCGGTGTCCACGCC
ΔP168 reverse	GACACCGGTGAGTTCCATATGGTGCATG
A173V forward	GTGTCCACGTAGGTACAGATCTGGAAGG
A173V reverse	CAGATCTGTACCTACGTGGACACCGG
P252L forward	GATATCCTGGGTCTACTCAGTGCCCAGACAG
P252L reverse	CTGGGCACTGAGTAGACCCAGGATATCAACATG
T304I forward	AGTGGGGTCATCTTCCAGAGTGCAGTGAAAAGAAC
T304I reverse	CACTCTGGAAGATGACCCCACTGCATTGTCTGAC
Forward sequencing primer	CGCAAATGGGCGGTAGGCGTG
Reverse sequencing primer	TAGAAGGCACAGTCGAGG
